# Japanese Surveillance Systems and Treatment for Influenza

**DOI:** 10.1007/s40506-016-0085-5

**Published:** 2016-10-10

**Authors:** Hassan Zaraket, Reiko Saito

**Affiliations:** 1 Department of Experimental Pathology, Immunology and Microbiology, Faculty of Medicine, American University of Beirut, Beirut, Lebanon; 2 Center for Infectious Diseases Research, Faculty of Medicine, American University of Beirut, Beirut, Lebanon; 3 Division of International Health, Graduate School of Medical and Dental Sciences, Niigata University, 1-757, Asahimachi-dori, Chuo-ku, Niigata City, Niigata Prefecture 951-8510 Japan

**Keywords:** Influenza, Surveillance systems, Epidemiology, Pathogen surveillance, Antiviral treatment, Neuraminidase inhibitors, Oseltamivir, Zanamivir, Laninamivir, Peramivir, Favipiravir

## Abstract

Influenza management and surveillance programs in Japan possess several unique features. The national influenza surveillance is affiliated with National Epidemiological Surveillance for Infectious Diseases (NESID) and features sentinel outpatient surveillance, virological surveillance, and reports on hospitalization, mortality, and influenza-associated encephalopathy. Of note, information on the number of student absences and class/grade/school closures due to influenza are also reported to the government and made publically available. A private online influenza surveillance portal by volunteer doctors provides a real-time information source for the Japanese clinicians and the general public. For influenza treatment, three classes of drugs are approved and covered by national medical insurance in Japan: M2 inhibitors, neuraminidase inhibitors (NAIs), and a polymerase inhibitor. Four NAIs, oseltamivir, zanamivir, laninamivir, and peramivir, are licensed in Japan and are prescribed to seven to eight million patients annually. NAIs are prescribed to any influenza outpatient rather than being limited to severe cases. The majority (80–95 %) of patients start the treatment within 48 h of onset. Laninamivir and peramivir were used almost solely in Japan, until the approval of the latter drug by the FDA. Observational studies showed that the two drugs have equal effectiveness as oseltamivir and zanamivir. The Japanese approach to influenza surveillance and management has facilitated bringing new influenza antivirals to the markets and has driven innovative research in this field. New classes of antivirals, including polymerase inhibitors and cap-dependent endonuclease inhibitor, provide novel tools for treatment of influenza in Japan and the rest of the world.

## Introduction

Influenza is a highly transmissible viral infection associated with serious public health and economic issues. Each year, various strains of influenza viruses circulate throughout the world and cause significant morbidity and mortality [1]. So far, three types and subtypes of influenza virus, influenza A (H1N1) pandemic 2009 (pdm09), A(H3N2), and B are mainly associated with human infections [2]. Effective surveillance and monitoring of influenza outbreaks are critical for evaluating the impact of the disease on the community and for devising disease management policies. Japan has robust and unique national influenza surveillance systems comprising of countrywide, sentinel-based surveillance of influenza-like illness (ILI), viral surveillance, number of school absences, and excess mortality.

Vaccination is the most important measure for prevention of seasonal influenza, while antiviral drugs provide the cornerstone for early outbreak response in case of a pandemic [3]. Three neuraminidase inhibitors (NAIs) are currently licensed worldwide for treatment and prophylaxis of influenza infections: oseltamivir (Tamiflu®, Hoffmann-La Roche), zanamivir (Relenza®, GlaxoSmithKline), and peramivir (Rapiacta®, Shionogi or Rapivab, BioCryst) [4, 5•]. In addition to these three NAIs, laninamivir (Inavir®, Daiichi Sankyo) is exclusively available in Japan for influenza treatment and prophylaxis [6, 7]. Recently, Japan also approved a polymerase inhibitor, favipiravir (Avigan®), for treatment of influenza [8•, 9].

Japan is the one of top consumers of NAIs in the world [3, 6, 10, 11]. In recent years, the USA became the highest consumer of oseltamivir followed by Japan. During January to December 2014, Japan comprised 11.8 % of global sales, with the USA being the highest (71.5 %), Europe the third (7.7 %), and the rest of the world (9.0 %), according to the Media Release from F. Hoffmann-La Roche Ltd. [12].

In this review, we focus on the unique national influenza surveillance and clinical management and usage of neuraminidase inhibitors, such as laninamivir and peramivir that are not widely used in other countries. We also review new classes of anti-influenza drugs including favipiravir and S-033188.

## National Influenza Surveillance

### Outpatient Surveillance of Influenza-Like Illness

The infectious disease surveillance program in Japan, initiated in 1981, formed the basis for influenza surveillance for outpatients [13, 14]. This program was revised and updated to its present form following the revision of the Infectious Disease Control Law in 1999 [13–16]. The system is currently called National Epidemiological Surveillance for Infectious Diseases (NESID), which includes a mandatory reporting system for nationally notifiable diseases and sentinel surveillance systems for various kinds of infectious diseases [17].

Influenza falls under the sentinel surveillance arm of the program. Weekly numbers of influenza patients are reported from 5000 medical institutions nationwide to local health centers. Sentinel sites were designated according to their geographic distribution, type of medical institutions (clinic or hospital), and population densities. These sentinels use the following criteria for reporting ILI: (1) sudden onset of illness, (2) fever >38 °C, (3) symptoms of upper respiratory inflammation, and (4) systemic symptoms such as general fatigue. A case is considered to meet the reporting criteria if the patient meets all symptoms from (1) to (4) or with at least one of the symptoms combined with a positive rapid diagnostic test (RDT) [17]. Sentinel sites report the age group and sex of patients on a weekly basis. The report does not include personal information like names or addresses. This information is transferred from local health centers to the prefectural government where it is aggregated into a prefectural report. The report is then forwarded to the National Institute of Infectious Diseases (NIID) in Tokyo, which is affiliated to the Ministry of Health, Labour and Welfare (MHLW). Major cities report directly to NIID without going through the prefectural government. The data is aggregated at NIID into a weekly total number of cases and number of visits per medical institution at prefectural level. The surveillance reports are released on a weekly basis on the website of the Infectious Disease Weekly Report (http://www.nih.go.jp/niid/en/idwr-e.html).

Estimates from NESID data showed that the numbers of ILI cases presenting as outpatients during the 2009 pandemic and the four post-pandemic seasons (2009/2010, 2010/2011, 2011/2012, 2012/2013, and 2013/2014) were 20.9, 13.9, 16.8, 13.8, and 15.4 million, respectively [13, 14, 18]. It was estimated that almost 20 % of Japanese population was infected with the influenza A(H1N1)pdm09 at the start of pandemic in 2009, but this figure subsequently decreased in the following seasons.

### Virological Surveillance

Virological surveillance, consisting of testing for influenza virus and the analysis of viral antigenicity, is incorporated into outpatient surveillance under the scheme of NESID. Around 500 medical institutions out of 5000 sentinel sites are designated as pathogen collection sites [14]. Clinicians in the community routinely take nasopharyngeal swabs from ILI patients and send the samples to local (prefectural or municipal) public health laboratories. Currently, 81 public health laboratories are located in major municipalities and all 47 prefectures. Those public health laboratories are in charge of detecting influenza virus by isolation and PCR testing (a list of laboratories is available from web site of the Japan Association of Prefectural and Municipal Public Health Institute, URL at http://www.chieiken.gr.jp/, in Japanese only).

After initial typing and subtyping is performed, selected strains are sent to NIID for further analysis to be used for vaccine selection. Countrywide data including virus type and subtype is tabulated at NIID and made publicly available under the Infectious Agent Surveillance Report (IASR, http://www.nih.go.jp/niid/ja/iasr-inf.html). Around 6000–8000 influenza viruses are isolated annually in Japan. By combining ILI (NESID) and virological surveillance data, influenza type and subtype-specific [i.e., A(H1N1)pdm09, A(H3N2), and B] prevalence can be estimated [14].

### Influenza-Associated Hospitalizations

In addition to outpatient and virological surveillance, data on the number of influenza-associated hospitalizations is also collected under NESID. Around 500 hospitals nationwide possessing more than 300 beds are selected as sentinels. Reporting on hospitalization started in 2011. During the pandemic in 2009, physicians had to report all hospitalized cases with confirmed influenza A and influenza B via the internet-based National Epidemiological Surveillance of Infectious Diseases (iNESID). This web-based data collection system was made available only from July 24 to September 5, 2009 [16]. Data obtained from iNESID revealed that the overall hospitalization rate for influenza A(H1N1)pdm09 infection was 5.8 cases per 100,000 population; cases hospitalized for non-therapeutic purposes, such as mere quarantine, were excluded. The figure is almost one fourth of that which was reported from Australia and the USA [16], supporting the lower frequency of severe illness in Japan during the pandemic compared to other countries. So far, the NESID data on nationwide hospitalization rate of influenza after the first wave of the 2009 pandemic has not been published.

### Influenza-Associated Encephalopathy and Mortality

Influenza-associated encephalopathy (IAE) is an acute encephalopathy caused by influenza virus infection. IAE has been reported in Japan since late 1990s [19]. It is the leading cause of deaths associated with influenza infections among children in Japan. Nationwide reports on IAE started under the diseases entity of “acute encephalitis (AE)” since November 2003 under the NESID surveillance [20]. Case definitions of the IAE include (1) a positive result on viral culture, viral antigen testing, or viral ribonucleic acid polymerase chain reaction in respiratory specimens (or any organs) or a fourfold or greater rise in paired serum antibody titer test (hemagglutination inhibition or complement fixation test); (2) neurological signs and symptoms showing impaired consciousness, seizures, and abnormal behaviors and/or cognition. Impaired consciousness is defined as equal to or below 13 on the Glasgow Coma Scale. Abnormal behaviors and cognition are jumping from heights, hallucination, delirium, and agitation. In some cases, signs of increased intracranial pressure are observed, such as nausea, papillary edema, low blood pressure, abnormal ocular movement, or postures. These symptoms should persist for more than 24 h [21–24]; (3) abnormal brain CT or MRI findings that suggest mild encephalitis/encephalopathy with a reversible splenial lesion (MERS), one posterior reversible encephalopathy syndrome, and one diffuse swelling of the cerebrum and high signal areas in several regions of white or gray matter by diffusion-weighted image (DWI) or fluid-attenuated inversion recovery (FLAIR) [23].

Note that the detection of influenza virus from CSF is not required for a diagnosis of IAE in the Japanese guidelines because it has been shown that the influenza virus and inflammatory cells are not always detectable in the CSF of such patients, but they must have detectable influenza virus in their respiratory tract, and no other causes were found for this presentation [22•, 25–27].

The number of IAE cases increased by 6.9-fold during the 2009 pandemic compared to the previous seasons [28]. Of the 331 IAE cases reported in the year, 322 (97.2 %) were linked to influenza A(H1N1)pdm09 infection. The case fatality rate of IAE was 3.7 %, which was lower than that of influenza A- and B-associated encephalopathy reported in the previous seasons (12.9 and 14.0 %, respectively) [28].

Official records of the Ministry of Health, Labour and Welfare indicate 199 influenza-related deaths during the first wave of the 2009 pandemic (i.e., May 2009–May 2010) [29]. The mortality rate in Japan during the pandemic was estimated at 0.16 per 100,000 and is lower than that reported in other countries, such as the USA (3.96 per 100,000), Canada (1.32 per 100,000), Mexico (0.93 per 100,000), Australia (0.93 per 100,000), the UK (0.76 per 100,000), Singapore (0.57 per 100,000), Korea (0.53 per 100,000), and France (0.51 per 100,000) [29]. The variation in influenza mortality rates in different countries might be partly attributed to differences in the reporting systems and case definition in each country. However, overall these figures suggest lower mortality rate during the pandemic in Japan [30].

Sugaya reported that overall number of pediatric deaths during the first wave of the 2009 pandemic caused by the emergence of influenza A(H1N1)pdm09 virus was 38 among children 15 years old and under [31]. This is lower than the reported mortality figures for this age group during 2004–2008. It showed the pediatric deaths did not increase in Japan, in contrast to other countries such as the USA, which had a pediatric death rate several times higher in 2009.

The lower incidence of severe illness and lower mortality rates in Japan during the 2009 pandemic are believed to be a result of universal implementation of early treatment with NAIs in the community clinics [30, 31]. Early treatment with NAIs was more widely and more thoroughly implemented during the 2009 pandemic than ever before. A study of 1000 children hospitalized because of A(H1N1)pdm09 infection revealed that NAIs, primarily oseltamivir, had been used to treat 984 (98.4 %) of the 1000 patients [31]. Of note, 88.9 % (593/667) of the patients for whom accurate data were available, treatment with NAIs was initiated within 48 h after the onset of illness, and 69.9 % (466/667) of patients started treatment within 24 h after the onset of illness.

### Student Absence and School Closure Surveillance

Surveillance for student absence and school closures is a very unique feature of the Japanese influenza surveillance and public health intervention system. The School Health Act enacted in 1958 in Japan contains various regulations on school safety and hygiene [32]. The School Health and Safety Act in Japan states that children with influenza infection should stay home for at least 6 days after symptom onset [33]. The same act designates that the school masters can decide whether to close the class, grade, or school depending on the size of outbreaks.

The purpose of this regulation is to stop transmitting influenza among children and to minimize size of outbreaks at schools. This rule is applied not only to elementary schools but also to children’s day care centers, nursing schools, junior high schools, and high schools. Then those schools must report the number of absent students diagnosed with influenza as well as closures (class, grade, or school) to the local Education Board in each municipality [34].

The information is sent to the Ministry of Health, Welfare and Science, and the data is aggregated and released on the NIID website divided by prefectural or major city levels on a weekly basis (http://www.nih.go.jp/niid/ja/flu-flulike.html). Japanese class closure is generally a reactive measure. There is no nationwide uniform regulation on when to start class closures. It is up to school masters and the local Educational Board when to close, so the situation differs by school or area. In many cases, a class is closed when the number of absences due to ILI exceeds 10–20 %. Several groups evaluated the most optimal duration of class closure, and it was suggested that school closure lasting more than 4 or 5 days is effective to mitigate the spread of influenza [34–37].

### Volunteer-Based Web ILI Surveillance

In addition to surveillance by the public sector, there is a unique web-based outpatient surveillance, called ML-Influenza Database (http://ml-flu.children.jp/) [38]. This surveillance is organized by volunteer clinicians and allow real-time monitoring of ILI cases at outpatient clinics at a prefectural level. Currently, more than 200 doctors nationwide post the number of influenza patients diagnosed at their clinics or practice settings everyday through the ML-Influenza Database. The data is automatically updated and reflected on the web server. Site visitors can access nationwide influenza case numbers reported daily and the map with the ability to zoom in to city level.

A strong correlation (correlation coefficient = 0.81–0.99) has been demonstrated between the weekly number of ILI reported by NESID and that by ML-Influenza Database, indicating the high validity and usefulness of this volunteer-based surveillance [38]. Additional information such as influenza type screened by RDT, age group, male/female ratio, name of the RDT kit used, the anti-influenza drug prescribed, and the vaccination status of influenza patients are also available on the website. Since its launch in the 2000–2001 season, the ML-Influenza Database has been quite informative for clinicians and the general population as it allows real-time monitoring of influenza trends.

### Treatment of Influenza

Three classes of antivirals, M2 inhibitors, NAIs, and a RNA polymerase inhibitor, are approved and covered by national medical insurance in Japan. The M2 inhibitor, amantadine (Symmetrel®) is effective only for influenza A. However, the dug is rarely used in Japan in recent years because of the universal resistance among influenza A(H1N1)pdm09 and A(H3N2) attributed to an amino acid mutation serine to asparagine at position 31 in the M2 protein [39–42]. Thus, NAIs, which are effective against both influenza A and B, are now the drugs of choice for clinicians [3, 41] A newly developed RNA polymerase inhibitor, favipiravir (Avigan®), was approved in Japan in 2014, but the use is highly restricted due to its potential adverse effects [7, 9, 43].

### Treatment Guidelines for Outpatients

In terms of NAIs, four drugs are approved in Japan: oseltamivir (Tamiflu®), zanamivir (Relenza®), peramivir (Rapiacta®), and laninamivir (Inavir®). Zanamivir was approved in 1999, oseltamivir in 2000, and peramivir and laninamivir in 2010 [6]. Prescription of an NAI is approved to outpatients without any comorbidities, as well as to high-risk patients with multiple comorbidities.

Oseltamivir is administered orally for 5 days (2 × 75 mg/day for adults and children weighing >37.5 kg and 2 mg/kg/day for children <37.5 kg) (Table 1) [44, 45]. Zanamivir is administered by inhalation by patients daily for 5 days (2 × 10 mg/day for adults and for children ≥5 years) [46]. Laninamivir is administered by inhalation to adults and to children aged ≥10 years as a single dose of 40 mg (two devices) and to children <10 years old as single dose of 20 mg (one device), respectively [47]. Peramivir is intravenously infused once over a period of 15–30 min to infected adults at a dose of 300 mg and to children at 10 mg/kg as outpatients [48].

**Table 1:** Recommended dosage and schedule of influenza antiviral medications for treatment and chemoprophylaxis in Japan (as of 2016)

	Route of administration	Treatment		Chemoprophylaxis^a^	
		Adult	Children	Adult	Children
Oseltamivir	Oral	75 mg twice daily, 5 days	a. 75 mg twice daily, 5 days (body weight ≥ 37.5 kg)^b^ b. 2 mg/kg twice daily, 5 days (maximum 150 mg/day) (body weight < 37.5 kg)^b^	75 mg once daily, 10 days^c^	2 mg/kg once daily, 10 days (maximum 75 mg/day)^c^
Zanamivir	Inhalation	10 mg twice daily, 5 days	10 mg twice daily, 5 days (≥5 years old)	10 mg once daily, 10 days^d^	10 mg once daily, 10 days (≥5 years old)^d^
Peramivir	Intravenous infusion for 15–30 min	One 300 mg dose (maximum 600 mg per day), 1 day^e^	One dose at 10 mg/kg (maximum 600 mg/day), 1 day^e^	N/A	N/A
Laninamivir	Inhalation	One 40 mg dose, via inhalation	a. 40 mg single inhalation (≥10 years old) b. 20 mg single inhalation (<10 years old)	20 mg single inhalation daily, two successive days or o^c^	a. 20 mg single inhalation daily, two successive days, or one 40 mg single inhalation (≥10 years old) b. 20 mg single inhalation (<10 yeas old)^c^
Favipiravir	Oral	1600 mg twice daily as initial dose 600 mg twice daily for following 2–5 days	N/A	N/A	N/A

^a^Chemoprophylaxis is considered for family members or those sharing daily life with influenza patient and fulfill the conditions of (i) elderly over age of 65 years old, (ii) chronic respiratory diseases, and chronic cardiac diseases, (iii) metabolic disease such as diabetes mellitus, and (iv) renal dysfunction

^b^Administration to patients who aged 10–19 years is not recommended. Safety to children below 1 year old is not confirmed

^c^Recommended to start within 48 h after known contact with influenza patient

^d^Recommended to start within 1.5 days after known contact with influenza patient

^e^Successive daily administration is allowed for patients in severe condition

In Japan, governmental guidelines are not published but are rather included in the drug insert. The package inserts are frequently updated and describe detailed recommendations under the supervision of MHLW. To supplement package inserts, several academic societies, such as Japanese Association for Infectious Diseases [49], Japanese Society of Chemotherapy, Japan Pediatric Society, Japanese Respiratory Society, etc., issue recommendations and guidelines for the treatment of influenza for their specific group of patients. These guidelines are complementary of the governmental guidelines.

This approach to outpatient care for influenza is completely different from the situation in the European Union or the USA, where NAIs are mostly recommended for the higher risk groups (young children, elderly, or individuals of any age with underlying chronic illness) or residents of nursing homes or other long-term care facilities at risk of severe disease [4, 50].

The NAIs are generally effective when the treatment is initiated within the first 48–72 h after symptom onset [51–53]. In Japan, most of ambulatory patients start NAI treatment within 1–2 days of influenza onset. Studies have shown that 80–95 % of influenza patients in Japan visit a medical facility within 48 h of onset and started the treatment [54, 55]. Other studies have shown that earlier initiation of antiviral therapy is associated with improved clinical outcomes in all populations [52, 56–58].

Normally, NAIs are prescribed when an influenza RDT is shown to be positive [6]. The cost of the RDT is covered by national health insurance, which contributes to its widespread use. As of 2016, more than 20 kinds of RDT were available in the Japanese market. Clinicians can prescribe NAIs even if the RDT shows a negative result. The choice of which NAI to prescribe is solely up to the clinician and patient, except in the case of oseltamivir, where age restrictions do exist [7, 49, 59•]. Oseltamivir is not recommended for use to those aged 10 to 19 years old by the order of MHLW [60], following reports of abnormal behavior in teenagers after administration in 2007 [59•].

### Treatment Guidelines for Hospitalized Patients

Treatment with the NAIs is recommended for influenza patients who require hospital admission and are as follows: (1) life-threatening, complicated progressive illness: oseltamivir 75 mg twice daily for 5 days (excluding those aged 10–19 years old) or peramivir one 600-mg dose (repeated administration is allowed); (2) viral pneumonia: oseltamivir 75 mg twice daily for 5 days (excluding those aged 10–19 years old) or peramivir single 300-mg dose (600 mg per infusion when a patient is in severe condition and repeated administration is allowed); and (3) where illness is not life threatening and without the complication of pneumonia, all four NAIs are equally recommended. The dosage and duration of medication are the same as for influenza outpatients [49]. In general, peramivir is likely to be administered in severely ill patients or those who have complications because the concentration of the drug in blood is high and serial injections are possible [61].

### Numbers of NAI Doses Prescribed in Japan

Post-marketing surveys by the four manufacturers of NAIs, Chugai Pharmaceuticals, GlaxoSmithKline, Daiichi Sankyo, and Shionogi, were reported to the Ministry of Health, Labour and Welfare, allowing estimation of the number of NAI prescriptions between 2011 and 2015 influenza seasons [62–65].

**Fig. 1:**
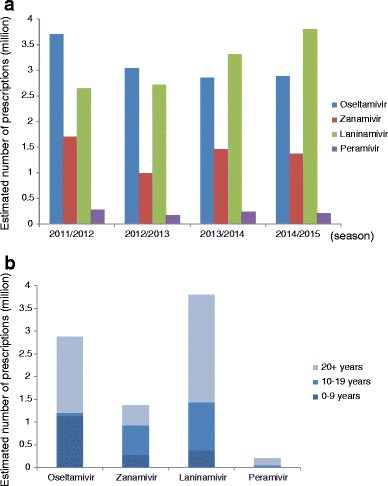
Estimated number of prescriptions for neuraminidase inhibitors (NAIs), oseltamivir, zanamivir, laninamivir, and peramivir, and age distribution of patients who received NAIs in Japan. **a** Estimated number of prescriptions of NAIs during four influenza seasons from 2011/12 to 2014/2015. **b** Age distribution during single season of 2014/2015 of patients who received NAIs

During the four influenza seasons, NAIs were prescribed to seven to eight million patients each year. Thus, more than half of influenza patients in Japan received NAIs. A vaccine effectiveness study revealed that 96 % of the influenza patients had received a prescription of NAI [54]. This data shows that prescriptions of anti-influenza agents are very common in Japan. Among the four agents, prescriptions of oseltamivir and laninamivir were the highest, with the two drugs accounting for 70–80 % of all NAIs (Fig. 1) [62–65].

Oseltamivir is easy to take because it uses the oral route as a capsule or syrup, so it is preferred for both pediatric and adult patients. In contrast, the two inhalation agents, zanamivir and laninamivir, are mainly prescribed for the teenage group. The prescription of the inhaled NAIs for children under 10 years old is expected to be lower due to difficulty of successful drug inhalation in this age group.

Laninamivir is a long-acting drug, and the treatment is completed in a single inhalation [47]. Due to its convenience, the sales of this drug are increasing year by year. Patients over 20 years old account for 62 % of laninamivir among all age groups. This shows that laninamivir is the preferred drug for adults and elderly (Fig. 1). In contrast, zanamivir is prescribed less frequently in patients over 20 years of age (32 %) because it requires multiple inhalations, twice daily for 5 days, which is less convenient for busy, working adults. Peramivir is administered via drip infusion, and it approximately accounts for 3 % of prescriptions among the four NAIs. Although peramivir is allowed to be used in outpatients with mild illness [48], there is an ethical dispute about intravenous (IV) administration for outpatients because a similar clinical effect is expected with the rest of three NAIs requiring less invasive administration. Thus, the administration of peramivir is mainly centered on hospitalized, critically ill patients, the elderly, and vomiting patients for whom oral intake is not feasible at outpatient clinics.

### Suspension of Oseltamivir Prescription to Teenagers

The suspension of oseltamivir for teenagers goes back to the incidence in 2007. In February 2007, two Japanese junior high students who were receiving oseltamivir for the treatment of influenza jumped from buildings and died [59•]. Oseltamivir was suspected to be the cause of the incidence, although a causal relationship between the treatment and incidents was not confirmed. In response to these events, the Dear Healthcare Professional Letters of Emergent Safety Communications (the Yellow Letter) was issued in March 2007 ordering clinicians to suspend the prescription of oseltamivir in influenza patients aged 10–19 years of age [11, 59•, 60].

Immediately after the announcement, the use of oseltamivir for 10–19 years age group dropped dramatically by 63.2 % [11], an indication of the significant impact of the governmental action. Later, delirious behaviors were also reported in patients receiving other NAIs [6, 59•, 66]. However, abnormal behaviors are generally observed during the early stage of influenza illness, within 48 h after the onset of illness [6]. This triggered investigations focused on studying the relationship between abnormal behaviors and oseltamivir or other NAI use in Japan. Currently, it is generally accepted that there is no causal relationship between oseltamivir or other NAI intake and abnormal behavior [6, 59•, 66, 67]. Yet, the government restriction on oseltamivir use in teenagers has not been lifted (Fig. 1).

### Laninamivir

Laninamivir (laninamivir octanonate, CS-8958) is currently only licensed in Japan. The drug can be used for outpatients as well as hospitalized patients if they can inhale the drug.

Laninamivir exhibits long-lasting neuraminidase inhibitory activity against influenza A and B viruses, including oseltamivir-resistant viruses [68]. The drug is retained in the respiratory tract for up to 6 days after a single inhalation [69, 70]. After the licensure, it is generally observed that laninamivir is as equally effective as the other three NAIs against influenza A(H1N1)pdm09, A(H3N2), and B to children and adults [71–75].

Owing to its single dose regimen, laninamivir’s sales exceeded those of oseltamivir becoming the top-selling NAI in Japan during the 2014–2015 season (Fig. 1). Of note, the convenience of laninamivir’s single inhaled administration could also be a disadvantage if the patient fails to inhale the dose properly, which is often the case in children younger than 10 years old. Therefore, the medical staff or pharmacists need to observe young children and assess whether they can inhale [76]. A special training device making a whistling sound is distributed free of charge from the manufacturer [76].

In Japan, laninamivir is approved for prophylaxis of influenza. Initial randomized double controlled trial showed that relative risk reductions in secondary family infections were 77.0 % for those given 20 mg of laninamivir once daily for 2 days and 78.1 % for those on 3-day regimen compared to the placebo [77]. Based on these findings, laninamivir was approved for prophylaxis at a dose of 20 mg for two successive days for patients ≥10 years old (Table 1). A recent randomized double-blind controlled trial demonstrated the effectiveness of single 40 mg laninamivir inhalation in post-exposure prophylaxis against influenza in adults [78•]. As of 28 August 2016, in a modified prophylaxis regimen, but the same as treatment course, a single inhalation of 40 mg for children aged ≥10 years and adults and one inhalation of 20 mg for children <10 years were added together with separated 2-day doses previously applied (press release from Daiichi Sankyo is available at http://www.daiichisankyo.co.jp/news/detail/006494.html, Japanese only).

### Peramivir

Peramivir is an IV NAI that has been only approved for the treatment (but not prophylaxis) of influenza in Japan since 2010 [5, 6, 79]. In the USA, the drug was temporarily approved on November 19, 2009 by Emergency Use Authorization (EUA) by the Commissioner of the Food and Drug Administration [80]. Its use has been limited to hospitalized patients with laboratory confirmed influenza A(H1N1)pdm09 infection who did not respond to oral or inhaled antiviral therapy [81]. In 2015, peramivir was fully licensed by the Food and Drug Administration (FDA) in the USA [82]. In South Korea, peramivir has also been available in patients with pandemic A(H1N1)pdm09 virus from November 2009 and has been authorized for sale since August 2010 [83]. The drug was also used in China for treatment of severe human infections caused by influenza A(H7N9) in 2013 [84].

Clinical trials in Japan demonstrated that 300 and 600 mg IV administration of peramivir significantly reduced the time to the alleviation of symptoms compared to placebo [85]. A phase III study in Korea, Japan, and Taiwan also showed that the time to the alleviation of symptoms upon a single 300 or 600 mg IV peramivir dose was comparable to orally administrated oseltamivir [86].

Peramivir is approved for children at 10 mg/kg (600 mg maximum dose) [87]. In high-risk patients, the use of multiple peramivir doses is also licensed in Japan [61]. In contrast, in the USA, peramivir is only approved for acute uncomplicated influenza in patients 18 years and older who have been symptomatic for no more than 2 days [5].

Peramivir is approved in Japan for ambulatory patients without complications, but the drug is not prescribed as frequently as oseltamivir, zanamivir, and laninamivir. The estimated prescription rate of peramivir in Japan was around 200,000 doses in the recent four seasons from 2011 to 2015, which is almost one tenth of other three NAIs (Fig. 1). Peramivir is mainly administered in adults and elderly (76 %) (Fig. 1), who are admitted to hospital and able to tolerate intravenous administration. Limited observational studies are available for peramivir after licensure due to the relatively low prescription rate compared to the other NAIs [71, 72, 74, 88–91].

Ambulatory children infected with influenza A(H1N1)pdm09 [72] or A(H3N2) [71] had faster alleviation of fever with peramivir compared to the other NAIs. Adult patients diagnosed with influenza A and B also reported faster alleviation of fever by peramivir compared to zanamivir and oseltamivir [74]. Other studies have reported a similar level of effectiveness of peramivir compared to the other three NAIs in ambulatory adults and children with confirmed influenza A or B infections [88, 89]. Sato et al. reported that fever duration after a single IV dose of peramivir did not differ between influenza A- and B-infected patients, but the viral load decreased faster in the former group [90]. For hospitalized patients, there were no significant differences between the peramivir and oseltamivir groups with respect to the time to fever alleviation [91].

One of the important virological issues regarding peramivir treatment is the emergence of drug resistance. The most commonly detected H274Y (H275Y in N1 numbering) mutation in the NA gene confers cross-resistance to oseltamivir and peramivir [41, 92, 93]. Only one report is available at the moment regarding the efficacy of peramivir treatment against H275Y mutant A(H1N1)pdm09 virus. Kakuya et al. demonstrated in a small number of pediatric patients that oseltamivir and peramivir can retain some clinical effectiveness against the H275Y mutant virus, in a community outbreak in Hokkaido, Japan during the 2013–2014 season [94]. To retain the optimal concentration in blood and the upper respiratory tract in these children, two IV peramivir doses per day (5 or 10 mg/kg at every 12 h) are preferable based on the PK/PD dynamic models [90]. This course does not follow the guidelines in Japan, so clinicians need to obtain an informed consent from the patient prior to initiating the treatment. While most of hospitals possess a stock of peramivir, it is still less frequently used compared to other NAIs, makes observational studies difficult. Therefore, multi-center observational studies are needed to more accurately evaluate the efficacy of peramivir in severely ill patients.

### Novel Anti-influenza Drugs in Japan

Favipiravir, T-705, is a selective inhibitor of the RNA-dependent RNA polymerase of influenza virus and is also reported to inhibit a broad range of other RNA viruses [95–97]. Additionally, since it does not target the NA, it is effective against NAI-resistant influenza viruses [98]. A phase III clinical trial of favipiravir, a double-blind, randomized study comparing it against oseltamivir has been conducted in Japan since 2009 [99]. Favipiravir was reported to show non-inferiority compared to oseltamivir [7], but the results have not yet been published in the international peer-reviewed literature. Although favipiravir has been approved in Japan since 2014, its use is restricted to occasions when the government issues a special permit, such as in the case of infection with a new influenza subtype or when an influenza virus with reduced effectiveness to the other licensed NAIs is observed [9].

Teratogenicity and embryotoxicity in animal experiments has also been observed with favipiravir, which restricted its use for the treatment of influenza in everyday practice [7, 9]. However, the animal model demonstrated that favipiravir effectively protects mice from lethal infection with oseltamivir-sensitive or oseltamivir-resistant highly pathogenic H5N1 viruses [100]. In addition, the drug has been shown to be a promising treatment for Ebola virus infections [9, 101]. Therefore, the drug can be useful in the treatment of some severe viral infections.

Another new class of anti-influenza drug is currently in clinical trials in Japan. The drug, S-033188, is a novel RNA polymerase inhibitor and cap-dependent endonuclease inhibitor developed by Shionogi Pharmaceutical Co., Ltd. [102]. Phase II clinical trials began in October 2015 and were completed in 2016. Phase III clinical trials in multiple countries are planned to commence during the 2016/2017 season.

### Summary

In summary, the approach to influenza in terms of clinical management and public health surveillance in Japan exhibits several novel features. This has driven innovative antiviral drug development in the country, which has benefited other countries in the form of alternative drug therapies for the treatment of this ever-changing virus. The extensive and large-scale surveillance of influenza and ILI in Japan also encouraged the high consumption of anti-influenza drugs raising concerns about its potential to select resistant viruses.

## References

[1] Neuzil KM, Mellen BG, Wright PF, Mitchel EF, Griffin MR (2000). The effect of influenza on hospitalizations, outpatient visits, and courses of antibiotics in children. N Engl J Med.

[2] World Health Organization (2016). Recommended composition of influenza virus vaccines for use in the 2016–2017 northern hemisphere influenza season. Wkly Epidemiol Rec.

[3] Nguyen-Van-Tam JS, Venkatesan S, Muthuri SG, Myles PR (2015). Neuraminidase inhibitors: who, when, where?. Clin Microbiol Infect.

[4] Centers for Disease Prevention and Control. Influenza antiviral medications: summary for clinicians. Available from: http://www.cdc.gov/flu/professionals/antivirals/summary-clinicians.htm (Accessed on 12 June 2016).

[5] • Alame MM, Massaad E, Zaraket H. Peramivir: a novel intravenous neuraminidase inhibitor for treatment of acute influenza infections. Front Microbiol. 2016;7. This is a good review for both researchers and clinicians regarding current updates for peramivir. It covers drug design, mode of action, mechanism and current situation on drug resistance, the latest information on side effects, results of clinical trials and post-marketing surveys. 10.3389/fmicb.2016.00450 PMC4815007 27065996

[6] Sugaya N (2011). Widespread use of neuraminidase inhibitors in Japan. J Infect Chemother.

[7] Sugaya N. [Antiviral treatment for influenza]. Influenza clinical managemant guidebook, 2015–2016. 2015:1–239, Japanese.

[8] Takashita E, Ejima M, Ogawa R, Fujisaki S, Neumann G, Furuta Y (2016). Antiviral susceptibility of influenza viruses isolated from patients pre- and post-administration of favipiravir. Antivir Res.

[9] Nagata T, Lefor AK, Hasegawa M, Ishii M (2015). Favipiravir: a new medication for the Ebola virus disease pandemic. Disaster Med Public Health Prep.

[10] Takashita E, Fujisaki S, Shirakura M, Nakamura K, Kishida N, Kuwahara T (2016). Influenza A(H1N1)pdm09 virus exhibiting enhanced cross-resistance to oseltamivir and peramivir due to a dual H275Y/G147R substitution, Japan, March 2016. Eurosurveillance.

[11] Hanatani T, Sai K, Tohkin M, Segawa K, Antoku Y, Nakashima N (2014). Evaluation of two Japanese regulatory actions using medical information databases: a 'Dear Doctor' letter to restrict oseltamivir use in teenagers, and label change caution against co-administration of omeprazole with clopidogrel. J Clin Pharm Ther.

[12] F. Hoffmann-La Roche Ltd. Media Release. Roche delivers solid results in 2014. . Available from: http://www.roche.com/med-cor-2015-01-28-e.pdf (Accessed on 12 June 2016).

[13] Nakamura Y, Sugawara T, Kawanohara H, Ohkusa Y, Kamei M, Oishi K (2015). Evaluation of estimated number of influenza patients from national sentinel surveillance using the national database of electronic medical claims. Jpn J Infect Dis.

[14] Murakami Y, Hashimoto S, Kawado M, Ohta A, Taniguchi K, Sunagawa T (2016). Estimated number of patients with influenza A(H1)pdm09, or other viral types, from 2010 to 2014 in Japan. PLoS One.

[15] Okabe N, Yamashita K, Taniguchi K, Inouye S (2000). Influenza surveillance system of Japan and acute encephalitis and encephalopathy in the influenza season. Pediatr Int.

[16] Shimada T, Sunagawa T, Taniguchi K, Yahata Y, Kamiya H, Yamamoto KU (2015). Description of hospitalized cases of influenza A(H1N1)pdm09 infection on the basis of the national hospitalized-case surveillance, 2009-2010. Japan Jpn J Infect Dis.

[17] Health Service Bureau Ministry of Health Labour and Welfare. [The guidelines for National Epidmeiological Surveillance of Infectious Diseases: Influenza]. Available from: http://www.mhlw.go.jp/bunya/kenkou/kekkaku-kansenshou11/01–05-28.html (accessed on 30 July 2016) Japanese.

[18] Kawato M, Hashimoto S, Murakami Y, Ohta A, Taniguchi K, Sunagawa T, et al. [Evaluation of National Epidemiological Surveillance for Infectious Diseases (NESID) and esimate of numer of cases nationwide]. Annual Report of Study Group for "Enforcement of Infectious Disease Surveillance and Risk Assessment for Emerging and Reemerging DiseasesSurveillance". Grant in-Aid by the Ministry of Health, Labor and Welfare, Health and Labor Sciences Research Grants, Japan 2016:68–82 Japanese.

[19] Morishima T, Togashi T, Yokota S, Okuno Y, Miyazaki C, Tashiro M (2002). Encephalitis and encephalopathy associated with an influenza epidemic in Japan. Clin Infect Dis.

[20] National Institute of Infectious Diseases Ministry of Health Labour and Welfare. [Acute encephalitis in Japan, January 2004–August 2007]. Infectious Agents Surveillance Report. Available from: http://idsc.nih.go.jp/iasr/28/334/tpc334.html. (accessed on 30 July 2016) Japanese 2007;28:339–340.

[21] Nagao T, Morishima T, Kimura H, Yokota S, Yamashita N, Ichiyama T (2008). Prognostic factors in influenza-associated encephalopathy. Pediatr Infect Dis J.

[22] Mizuguchi M (2013). Influenza encephalopathy and related neuropsychiatric syndromes. Influenza Other Respir Viruses.

[23] Kawashima H, Morichi S, Okumara A, Nakagawa S, Morishima T (2012). National survey of pandemic influenza A (H1N1) 2009-associated encephalopathy in Japanese children. J Med Virol.

[24] Morishima T, Okabe N, Namakura Y, Kawaoka Y, Yamaguchi S, Mizuguchi M, et al. [Guideline for influenza encephalopathy: revised edition]. Jpn J Pediatr 2009;62:2483–2528 Japanese.

[25] Fujimoto S, Kobayashi M, Uemura O, Iwasa M, Ando T, Katoh T (1998). PCR on cerebrospinal fluid to show influenza-associated acute encephalopathy or encephalitis. Lancet.

[26] Kondo M (2003). A bad dose of the 'flu. Lancet.

[27] Weitkamp JH, Spring MD, Brogan T, Moses H, Bloch KC, Wright PF (2004). Influenza A virus-associated acute necrotizing encephalopathy in the United States. Pediatr Infect Dis J.

[28] Gu Y, Shimada T, Yasui Y, Tada Y, Kaku M, Okabe N (2013). National Surveillance of influenza-associated encephalopathy in Japan over six years, before and during the 2009-2010 influenza pandemic. PLoS One.

[29] Pandemic Influenza Prevention and Control Headquarters Ministry of Health Labour and Welfare. [Situation updates of pandemic influenza; country data, 6th Meeting for Management and Control of Pandemic Influenza, Ministry of Health, Labour and Welfare (28 May 2010)]. 2010:Available from: http://www.mhlw.go.jp/bunya/kenkou/kekkaku-kansenshou04/dl/infu100528–05.pdf (accessed on 18 Aug 2016) Japanese.

[30] Kamigaki T, Oshitani H (2009). Epidemiological characteristics and low case fatality rate of pandemic (H1N1) 2009 in Japan. PLoS Curr.

[31] Sugaya N, Shinjoh M, Mitamura K, Takahashi T (2011). Very low pandemic influenza A (H1N1) 2009 mortality associated with early neuraminidase inhibitor treatment in Japan: analysis of 1000 hospitalized children. J Infect.

[32] Takayama K (2010). Recent tasks of school health administration in Japan. Journal of the Japan Medical Association.

[33] Kondo H, Shobugawa Y, Hibino A, Yagami R, Dapat C, Okazaki M (2016). Influenza virus shedding in laninamivir-treated children upon returning to school. Tohoku J Exp Med.

[34] Kawano S, Kakehashi M (2015). Substantial impact of school closure on the transmission dynamics during the pandemic flu H1N1-2009 in Oita. Japan PLoS One.

[35] Hasui M, Yamagami M (2012). [A study of the duration of class closure during influenza A/HINI 2009 outbreaks in 2009–2010.]. The Journal of Ambulatory and General Pediatrics.

[36] Eda K, Otaguro S, Matsushima T, Shinagawa A, Ikematsu H, Kashiwagi S (2012). [Epidemiological study of A (H1N1) pdm09 in Iki Island, Nagasaki]. Kansenshogaku Zasshi.

[37] Sugiura H, Hata T, Kodama K, Okikawa K, Imamura T, Ohkusa Y (2010). [Examination of the effectiveness of class closure for A/H1N1(2009) pdm using the automatic information sharing system for school absentees]. Japanese Journal of School Health.

[38] Saito N. [ML Influenza Database. Web-based real-time monitoring systems for influenza epidemics in Japan.]. Annual Report of Study Group for "Enforcement of Infectious Disease Surveillance and Risk Assessment for Emerging and Reemerging DiseasesSurveillance". Grant in-Aid by the Ministry of Health, Labor and Welfare, Health and Labor Sciences Research Grants, Japan 2016:121–136 Japanese.

[39] Dapat IC, Dapat C, Baranovich T, Suzuki Y, Kondo H, Shobugawa Y (2012). Genetic characterization of human influenza viruses in the pandemic (2009-2010) and post-pandemic (2010-2011) periods in Japan. PLoS One.

[40] Zaraket H, Kondo H, Hibino A, Yagami R, Odagiri T, Takemae N, et al. Full genome characterization of human influenza A/H3N2 isolates from Asian countries reveals a rare amantadine resistance-conferring mutation and novel PB1-F2 polymorphisms. Front Microbiol. 2016;7. 10.3389/fmicb.2016.00262 PMC4779883 27014195

[41] Hurt AC (2014). The epidemiology and spread of drug resistant human influenza viruses. Current Opinion in Virology.

[42] Oh DY, Hurt AC (2014). A review of the antiviral susceptibility of human and avian influenza viruses over the last decade. Scientifica.

[43] Fuji Film Toyama Pharmaceuticals. [Media release. Approval of antiinfluenza drug "Avigan 200 mg tablet" in Japan]. Available from: https://www.toyama-chemical.co.jp/news/detail/140324.html (Accessed on 12 June 2016) Japanese.

[44] Chugai Pharmaceuticals Co.Ltd. [Package insert of Tamiflu capsule 75 mg]. Available from: http://www.info.pmda.go.jp/go/pack/6250021M1027_1_32/ (Accessed on 7 June 2016) Japanese.

[45] Chugai Pharmaceuticals Co.Ltd. [Package insert of Tamiflu DrySyrup 3 %]. Available from: http://www.info.pmda.go.jp/go/pack/6250021R1024_1_24/ (Accessed on 7 June 2016) Japanese.

[46] GlaxoSmithKline. [Package insert of Relenza]. Available from :http://www.info.pmda.go.jp/go/pack/6250702G1028_1_18/ (Accessed on 7 June 2016) Japanese.

[47] Daiichi-Sankyo Co.Ltd. [Package insert of Inavir dry powder inhaler 20 mg]. Available from :http://www.info.pmda.go.jp/go/pack/6250703G1022_1_12/ (Accessed on 7 June 2016) Japanese.

[48] Shionogi Pharmaceutical Co.Ltd. [Package insert of Rapiacta Bag 300 mg for intravenous drip infusion]. Available from :http://www.info.pmda.go.jp/go/pack/6250405A1032_1_03/ (Accessed on 7 June 2016) Japanese.

[49] Working Group for Treatment Guidelines of Infectious Disease-Japanese Association for Infectious Disease/Japanese Society for Chemohterapy. [JAID/JSC treatment and management guidelines for infectious disease—respiratory infectious diseases]. Japanese Journal of Chemotherapy 2014(62):1–109 Japanese.

[50] Penttinen P, Catchpole M (2016). ECDC expert opinion on efficacy and effectiveness of neuraminidase inhibitors published for public consultation. Influenza Other Respir Viruses.

[51] Moscona A (2005). Drug therapy—neuraminidase inhibitors for influenza. N Engl J Med.

[52] Aoki FY, Macleod MD, Paggiaro P, Carewicz O, El Sawy A, Wat C (2003). Early administration of oral oseltamivir increases the benefits of influenza treatment. J Antimicrob Chemother.

[53] Fry AM, Goswami D, Nahar K, Sharmin AT, Rahman M, Gubareva L (2014). Efficacy of oseltamivir treatment started within 5 days of symptom onset to reduce influenza illness duration and virus shedding in an urban setting in Bangladesh: a randomised placebo-controlled trial. Lancet Infect Dis.

[54] Shinjoh M, Sugaya N, Yamaguchi Y, Tomidokoro Y, Sekiguchi S, Mitamura K (2015). Effectiveness of trivalent inactivated influenza vaccine in children estimated by a test-negative case-control design study based on influenza rapid diagnostic test results. PLoS One.

[55] Ikematsu H, Kawai N, Iwaki N, Kashiwagi S (2015). Clinical outcome of laninamivir octanoate hydrate for influenza in the 2013-2014 Japanese season. J Infect Chemother.

[56] Whitley RJ, Hayden FG, Reisinger KS, Young N, Dutkowski R, Ipe D (2001). Oral oseltamivir treatment of influenza in children. Pediatr Infect Dis J.

[57] Cooper NJ, Sutton AJ, Abrams KR, Wailoo A, Turner DA, Nicholson KG (2003). Effectiveness of neuraminidase inhibitors in treatment and prevention of influenza A and B: systematic review and meta-analyses of randomised controlled trials. Br Med J.

[58] Muthuri SG, Myles PR, Venkatesan S, Leonardi-Bee J, Nguyen-Van-Tam JS (2013). Impact of neuraminidase inhibitor treatment on outcomes of public health importance during the 2009-2010 influenza A(H1N1) pandemic: a systematic review and meta-analysis in hospitalized patients. J Infect Dis.

[59] Nakamura Y, Sugawara T, Ohkusa Y, Taniguchi K, Miyazaki C, Momoi M (2015). Life-threatening abnormal behavior incidence in 10-19 year old patients administered neuraminidase inhibitors. PLoS One.

[60] Pharmaceutical Affairs and Food Sanitation Council of the Ministry of Health Labour and Welfare. [Abnormal behavior after administrated Tamiflu. Emergency release of The Dear Healthcare Professional Letters of Emergent Safety Communications (Yellow Letter) about Tamiflu]. Available from: http://www.mhlw.go.jp/houdou/2007/03/h0320–1.html (accesed on 30 July 2016) Japanese 2007.

[61] Kohno S, Kida H, Mizuguchi M, Hirotsu N, Ishida T, Kadota J (2011). Intravenous peramivir for treatment of influenza A and B virus infection in high-risk patients. Antimicrob Agents Chemother.

[62] Subcommittee on Drug Safety of Committee on Drug Safety in the Pharmaceutical Affairs and Food Sanitation Council of the Ministry of Health Labour and Welfare. [4th Investigation Committee for Safety, Reference materials 3–2. Usages of anti-influenza virus drugs (Kou influenza virus yaku no siyoujoukyou)]. Available from : http://www.mhlw.go.jp/file/05-Shingikai-11121000-Iyakushokuhinkyoku-Soumuka/0000035794.pdf (Accessed on 8 June 2016) Japanese 2012.

[63] Subcommittee on Drug Safety of Committee on Drug Safety in the Pharmaceutical Affairs and Food Sanitation Council of the Ministry of Health Labour and Welfare. [5th Investigation Committee for Safety,Reference materials 2–2. Usages of anti-influenza virus drugs (Kou influenza virus yaku no siyoujoukyou).]. Available from : http://www.mhlw.go.jp/file/05-Shingikai-11121000-Iyakushokuhinkyoku-Soumuka/0000035672.pdf (Accessed on 8 June 2016) Japanese 2013.

[64] Subcommittee on Drug Safety of Committee on Drug Safety in the Pharmaceutical Affairs and Food Sanitation Council of the Ministry of Health Labour and Welfare. 6th Investigation Committee for Safety, Reference materials 2–2. Usages of anti-influenza virus drugs (Kou influenza virus yaku no siyoujoukyou). Available: http://www.mhlw.go.jp/file/05-Shingikai-11121000-Iyakushokuhinkyoku-Soumuka/0000063406.pdf Japanese (Accessed on 8 June 2016) 2014.

[65] Subcommittee on Drug Safety of Committee on Drug Safety in the Pharmaceutical Affairs and Food Sanitation Council of the Ministry of Health Labour and Welfare. [7th Investigation Committee for Safety, Reference materials 2. Usages of anti-influenza virus drugs (Kou influenza virus yaku no siyoujoukyou).]. Available from: http://www.mhlw.go.jp/file/05-Shingikai-11121000-Iyakushokuhinkyoku-Soumuka/0000063406.pdf (Accessed on 8 June 2016) Japanese 2015.

[66] Nakamura Y, Sugawara T, Ohkusa Y, Taniguchi K, Miyazaki C, Momoi M (2014). Abnormal behavior during influenza in Japan during the last seven seasons: 2006-2007 to 2012-2013. J Infect Chemother.

[67] Yokota S, Fujita T, Mori M, Nezu A, Okumura A, Hosoya M (2007). [Epidemiologic survey of influenza associated complications I. Clinical assessment of symptoms and signs, and medication]. Nihon Syounikagakkai Zatsushi.

[68] Watanabe A, Chang SC, Kim MJ, Chu DWS, Ohashi Y, Grp MS (2010). Long-acting neuraminidase inhibitor laninamivir octanoate versus oseltamivir for treatment of influenza: a double-blind, randomized. Noninferiority Clinical Trial Clinical Infectious Diseases.

[69] Ishizuka H, Yoshiba S, Okabe H, Yoshihara K (2010). Clinical pharmacokinetics of laninamivir, a novel long-acting neuraminidase inhibitor, after single and multiple inhaled doses of its prodrug, CS-8958, in healthy male volunteers. J Clin Pharmacol.

[70] Yamashita M, Hirai T, Kubota K, Kubo S (2011). Unique characteristics of long-acting neuraminidase inhibitor laninamivir octanoate (CS-8958) that explains its long-lasting activity. Influenza Other Respir Viruses.

[71] Shobugawa Y, Saito R, Sato I, Kawashima T, Dapat C, Dapat IC (2012). Clinical effectiveness of neuraminidase inhibitors-oseltamivir, zanamivir, laninamivir, and peramivir-for treatment of influenza A(H3N2) and A(H1N1)pdm09 infection: an observational study in the 2010-2011 influenza season in Japan. J Infect Chemother.

[72] Sugaya N, Sakai-Tagawa Y, Bamba M, Yasuhara R, Yamazaki M, Kawakami C (2015). Comparison between virus shedding and fever duration after treating children with pandemic A H1N1/09 and children with A H3N2 with a neuraminidase inhibitor. Antivir Ther.

[73] Mizuno T, Mizuno S, Kanda T (2014). Effects of vaccination and the new neuraminidase inhibitor, laninamivir, on influenza infection. PLoS One.

[74] Takemoto Y, Asai T, Ikezoe I, Yano T, Ichikawa M, Miyagawa S (2013). Clinical effects of oseltamivir, zanamivir, laninamivir and peramivir on seasonal influenza infection in outpatients in Japan during the winter of 2012-2013. Chemotherapy.

[75] Kawai N, Ikematsu H, Kawashima T, Maeda T, Ukai H, Hirotsu N (2013). Increased symptom severity but unchanged neuraminidase inhibitor effectiveness for A(H1N1)pdm09 in the 20102011 season: comparison with the previous season and with seasonal A(H3N2) and B. Influenza Other Respir Viruses.

[76] Kashiwagi S, Yoshida S, Yamaguchi H, Mitsui N, Tanigawa M, Shiosakai K (2012). [Administration setting and status of inhalation of the long-acting neuraminidase inhibitor laninamivir octanoate hydrate in post-marketing surveillance]. Japanese Journal of Chemotherapy.

[77] Kashiwagi S, Yoshida S, Yamaguchi H, Mitsui N, Tanigawa M, Shiosakai K (2013). Clinical efficacy of long-acting neuraminidase inhibitor laninamivir octanoate hydrate in postmarketing surveillance. J Infect Chemother.

[78] Kashiwagi S, Watanabe A, Ikematsu H, Uemori M, Awamura S (2016). Long-acting neuraminidase inhibitor laninamivir octanoate as post-exposure prophylaxis for influenza. Clin Infect Dis.

[79] Yoshida R (2010). Peramivir: a novel neuraminidase (NA) inhibitor against influenza virus with a promising inhibitory activity of in vitro NA activity and in vivo viral replication. J Pharmacol Sci.

[80] Birnkrant D, Cox E (2009). The emergency use authorization of peramivir for treatment of 2009 H1N1 influenza. N Engl J Med.

[81] Hernandez JE, Adiga R, Armstrong R, Bazan J, Bonilla H, Bradley J (2011). Clinical experience in adults and children treated with intravenous peramivir for 2009 influenza a (H1N1) under an emergency IND program in the United States. Clin Infect Dis.

[82] McLaughlin MM, Skoglund EW, Ison MG (2015). Peramivir: an intravenous neuraminidase inhibitor. Expert Opin Pharmacother.

[83] Yoo JW, Choi SH, Huh JW, Lim CM, Koh Y, Hong SB (2015). Peramivir is as effective as oral oseltamivir in the treatment of severe seasonal influenza. J Med Virol.

[84] Hu YW, Lu SH, Song ZG, Wang W, Hao P, Li JH (2013). Association between adverse clinical outcome in human disease caused by novel influenza A H7N9 virus and sustained viral shedding and emergence of antiviral resistance. Lancet.

[85] Kohno S, Kida H, Mizuguchi M, Shimada J, Grp SCS (2010). Efficacy and safety of intravenous peramivir for treatment of seasonal influenza virus infection. Antimicrob Agents Chemother.

[86] Kohno S, Yen MY, Cheong HJ, Hirotsu N, Ishida T, Kadota J (2011). Phase III randomized, double-blind study comparing single-dose intravenous peramivir with oral oseltamivir in patients with seasonal influenza virus infection. Antimicrob Agents Chemother.

[87] Sugaya N, Kohno S, Ishibashi T, Wajima T, Takahashi T (2012). Efficacy, safety, and pharmacokinetics of intravenous peramivir in children with 2009 pandemic H1N1 influenza a virus infection. Antimicrob Agents Chemother.

[88] Nakano T, Shiosakai K (2014). Spread of viral infection to family members from influenza patients treated with a neuraminidase inhibitor. J Infect Chemother.

[89] Kawai N, Ikematsu H, Maeda T, Hirotsu N, Ukai H, Kawashima T (2015). Epidemic situation and effectiveness of anti-influenza drug in 2013-2014 season. Influenza.

[90] Sato M, Ito M, Suzuki S, Sakuma H, Takeyama A, Oda S (2015). Influenza viral load and peramivir kinetics after single administration and proposal of regimens for peramivir administration against resistant variants. Antimicrob Agents Chemother.

[91] Yoshino Y, Seo K, Koga I, Kitazawa T, Ota Y (2015). Clinical efficacy of peramivir in adult patients with seasonal influenza during the winter of 2012 in Japan. Clinical Respiratory Journal.

[92] Gubareva LV, Webster RG, Hayden FG (2001). Comparison of the activities of zanamivir, oseltamivir, and RWJ-270201 against clinical isolates of influenza virus and neuraminidase inhibitor-resistant variants. Antimicrob Agents Chemother.

[93] McKimm-Breschkin JL (2013). Influenza neuraminidase inhibitors: antiviral action and mechanisms of resistance. Influenza Other Respir Viruses.

[94] Kakuya F, Kinebuchi T, Fujiyasu H, Tanaka R, Okubo H, Kano H (2015). Clinical findings in 10 children with H275Y influenza A(H1N1)pdm09 virus infection. Pediatr Int.

[95] Furuta Y, Gowen BB, Takahashi K, Shiraki K, Smee DF, Barnard DL (2013). Favipiravir (T-705), a novel viral RNA polymerase inhibitor. Antivir Res.

[96] McKimm-Breschkin JL, Fry AM (2016). Meeting report: 4th ISIRV antiviral group conference: novel antiviral therapies for influenza and other respiratory viruses. Antivir Res.

[97] Oestereich L, Ludtke A, Wurr S, Rieger T, Munoz-Fontela C, Gunther S (2014). Successful treatment of advanced Ebola virus infection with T-705 (favipiravir) in a small animal model. Antivir Res.

[98] Tarbet EB, Vollmer AH, Hurst BL, Barnard DL, Furuta Y, Smee DF (2014). In vitro activity of favipiravir and neuraminidase inhibitor combinations against oseltamivir-sensitive and oseltamivir-resistant pandemic influenza A (H1N1) virus. Arch Virol.

[99] Toyama Chem Co. Ltd. [Start of phase III clinical trial for anti-influenza drug, T-705]. Available from: http://www.toyama-chemical.co.jp/cgi-bin/ja_prtpreview/print.cgi/news/detail/091029.html (accessed on 29 July 2016) Japanese 2009.

[100] Kiso M, Takahashi K, Sakai-Tagawa Y, Shinya K, Sakabe S, Le QM (2010). T-705 (favipiravir) activity against lethal H5N1 influenza A viruses. Proc Natl Acad Sci U S A.

[101] Mora-Rillo M, Arsuaga M, Ramirez-Olivencia G, de la Calle F, Borobia AM, Sanchez-Seco P (2015). Acute respiratory distress syndrome after convalescent plasma use: treatment of a patient with Ebola virus disease contracted in Madrid, Spain. Lancet Respiratory Medicine.

[102] Shionogi Pharmaceutical Co.Ltd. [Press release "License contract engaged with F.Hoffma-La Roche Ltd. on anti-influenza drug, S-033188"]. Avalable from: http://www.shionogi.co.jp/company/news/2016/qdv9fu000000v5q2-att/160229.pdf#search='%E5%A1%A9%E9%87%8E%E7%BE%A9+%E3%82%A4%E3%83%B3%E3%83%95%E3%83%AB' (accessed on 29 July, 2016) Japanese 2016.

